# Lumbago and Muscular Rheumatism

**Published:** 1901-03

**Authors:** 


### Lumbago and Muscular Rheumatism.

℞ Menthol.................................. 20 gr.
Salicylic acid............................. 1 ℥
Chloral hydrate..........................
Camphor..............................aa 40 gr.
Powd. capsicum......................... 90 gr.
Croton oil................................. 5 gtt.
Petrolatum................................ 2 ℥

Rub in vigorously, a small quantity at a time.

